# Intraoperative neurophysiological monitoring in surgery for intramedullary spinal cord lesions – workflow, setup and outcomes

**DOI:** 10.1007/s00701-025-06697-z

**Published:** 2025-10-24

**Authors:** Henrik Frisk, Gayane Margaryan, Ali Buwaider, Davit Sargsyan, Victor Gabriel El-Hajj, Tomas Majing, Aman Singh, Alexander Fletcher-Sandersjöö, Oscar Persson, Victor E. Staartjes, Jonas K. E. Persson, Erik Edström, Adrian Elmi-Terander

**Affiliations:** 1 Department of Clinical Neuroscience, Karolinska Institutet, 171 77 Stockholm, Sweden; 2 Department of Perioperative Medicine and Intensive Care (PMI), Karolinska University Hospital, Stockholm, Sweden; 3 Department of Neurophysiology, Karolinska University Hospital, Stockholm, Sweden; 4 Johnson & Johnson, Innovative Medicine, Translational Medicine and Early Development Statistics, Raritan, NJ USA; 5 Department of Surgical Sciences, Uppsala University, Uppsala, Sweden; 6 Department of Neurosurgery, Karolinska University Hospital, Stockholm, Sweden; 7 Capio Spine Center Stockholm, Löwenströmska Hospital, Stockholm, Sweden; 8 Machine Intelligence in Clinical Neuroscience & Microsurgical Neuroanatomy (MICN) Laboratory, Department of Neurosurgery, Clinical Neuroscience Center, University Hospital Zurich, University of Zurich, Zurich, Switzerland; 9 Department of Medical Sciences, Örebro University, Örebro, Sweden

**Keywords:** Intraoperative neurophysiological monitoring, Intramedullary spinal cord lesion, Intraoperative monitoring, Spine, Workflow, Functional outcome

## Abstract

**Objective:**

Gross total resection is strived for in intramedullary spinal cord lesion surgery. Intraoperative neurophysiological monitoring (IONM) is the gold standard, but there is no consensus on the optimal IONM workflow. This study details our institutional workflow.

**Methods:**

We retrospectively reviewed all adults who underwent intramedullary resection at Karolinska University Hospital, 2007–2021 (*n* = 70). Continuous multimodal IONM (somatosensory-evoked potentials (SSEP), motor-evoked potentials (MEP) and epidural D-waves) was conducted by an in-room neurophysiologist. Alarm thresholds were preset (≥ 50% SSEP amplitude drop/10% latency rise; ≥ 80% MEP reduction; ≥ 50% D-wave loss) and triggered a standardized four-step rescue protocol (halt manipulation, raise MAP to 80–90 mm Hg, topical papaverine, observation). Motor/sensory function, modified McCormick (mMC) grade, pain, and sphincter control were documented pre-operatively, at 3 months, and ≥ 12 months.

**Results:**

Seventy patients were included. Most harboured ependymoma (51%), hemangioblastoma (18%) and cavernoma (8.5%). A neurophysiologist was present during every procedure. A ≥ 50% intra-operative SSEP-amplitude decrease was not followed by a sensory deficit (OR:3.0, 95% CI 0.86–10.6; *p* = 0.085) or mMC deterioration (OR:1.6, 0.33–7.5; *p* = 0.57) at either short- or long-term follow-up. In contrast, complete SSEP loss markedly increased the risk of postoperative sensory deficit (3-months-OR:25.2, 4.7–135; *p* < 0.001; long-term-OR 11.0, 2.8–43.8; *p* < 0.001) and poorer mMC grade (3-months-OR:7.8, 2.0–31; *p* = 0.004; long-term-OR:11.0, 2.8–43.8; *p* < 0.001). Loss of MEPs predicted a decline in mMC at long-term follow-up (OR:4.0, 1.06–15.1; *p* = 0.041).

**Conclusions:**

Live data from continuous intraoperative neurophysiological monitoring, expertly interpreted in the OR, could potentially be used to make surgical and anesthesiologic adjustments with the goal of minimizing the risk of negative neurological outcomes. Significant associations were found between decreased or lost IONM signals and poorer sensorimotor function and mMC score at short- and long-term follow-up. Implementation of the IONM workflow is suggested in all intramedullary surgery.

## Introduction

Intramedullary spinal cord lesions are rare lesions originating from the spinal cord itself [4]. The most common subtypes are ependymomas, astrocytomas, and hemangioblastomas [20]. These lesions typically manifest with neuropathic pain, along with motor and sensory deficits corresponding to the affected spinal cord segments [8]. If left untreated, patients may experience worsening pain and progressive neurological decline, potentially leading to paraplegia or quadriplegia. Radical surgical resection remains the most effective treatment and is associated with improved long-term survival [6]. However, the location of these lesions often limits the ability to achieve radical resection without risking significant neurological complications.

Intraoperative neurophysiological monitoring (IONM), utilizing somatosensory-evoked potentials (SSEPs), motor-evoked potentials (MEPs), and the direct wave (D-wave), is considered the gold standard for monitoring neurological function during spinal surgeries aimed at maximal resection [2]. IONM provides real-time feedback to the surgeon during the procedure, helping to determine whether to radically resect the lesion or to leave residual tissue in order to preserve neurological function and achieve more favorable neurological outcome [2].

Despite its widespread use, there is no clear consensus on the most optimal workflow or setup for IONM. A common approach involves having a technician present in the operating room while a neurophysiologist supervises remotely, often overseeing multiple cases simultaneously [18]. Other configurations include having a technician in the operating room with a neurophysiologist nearby for consultation [16], or having the neurophysiologist physically present in the operating room throughout the procedure [23]. In some cases the operating surgeon is also the one monitoring the neurophysiological parameters [12]. At the study center, a customized set-up has been used for over a decade. This study aims to present our institution's integrated workflow for neurophysiological monitoring during intramedullary spinal lesion surgeries and to report on postoperative outcomes following the use of this monitoring protocol.

## Method

This retrospective, population-based study included all adult patients who underwent spinal cord surgery between 2007 and 2021 at the Karolinska University Hospital in Stockholm, Sweden. The Karolinska University Hospital is a publicly funded, tertiary-care center and the sole neurosurgical provider for a regional population of approximately 2.3 million. Clinical data were extracted from electronic medical records using the TakeCare software (CompuGroup Medical Sweden AB, Farsta, Sweden). The study protocol received ethical approval from the regional ethics review board (approval number: EPN 2016/1708–31).

## Workflow

### Anesthesia

Anesthesia was induced with Propofol, and muscle relaxation for endotracheal intubation was achieved using a single dose of Rocuronium® at 0.6 mg/kg. Total intravenous anesthesia was maintained throughout the surgery using a Target-Controlled Infusion (TCI) of Propofol® and Remifentanil®, with dosing guided by effect-site concentrations (Ce-values) targeted to the brain. The Modified Marsh model was employed for Propofol® administration, while the Minto model was used for Remifentanil®. To preserve the integrity of neurophysiological monitoring, no additional doses of Rocuronium® were administered following intubation.

A protective chewing block was placed in the patient’s mouth to prevent damage to the endotracheal tube and guard against unintentional biting resulting from muscle twitching during neurophysiological stimulation. Patient monitoring included continuous assessment of both invasive and non-invasive blood pressure, electrocardiography (ECG), peripheral oxygen saturation (SpO₂), and end-tidal carbon dioxide (EtCO₂) capnography, ensuring comprehensive physiological surveillance throughout the procedure.

### Patient preparation

With the patient initially positioned supine, electrodes for neurophysiological monitoring were placed on the front of the body. For surgeries involving the cervical or upper thoracic spine, a Mayfield head clamp was applied. Insulated (non-conducting) Mayfield pins were used. For subcervical procedures, the patient’s head instead rested on a pillow. After electrode placement, the patient was carefully transferred from one operating table to another and repositioned into the prone position. The surgical site was disinfected, and the patient draped according to local routine before the surgical procedure began.

### Monitoring during surgery

Intraoperative neurophysiological monitoring was conducted using the Cadwell Cascade IONM System for both stimulation and recordings.

Baseline SSEPs and MEPs were recorded at the start of the surgical procedure, before the first incision. Following completion of the laminectomy, intraoperative ultrasound was performed to define the cranial and caudal extent of the lesion. D-wave electrodes were placed in the epidural space, cranial and caudal to the lesion, to facilitate intraoperative monitoring.

### Somatosensory evoked potentials (SSEPs)

For SSEP recording, corkscrew electrodes are placed on the scalp at four designated sites (Fz', Cz', C3', and C4') in accordance with the international 10–20 EEG system. Needle electrodes are positioned bilaterally over the brachial plexus. Electrical stimulation is administered using disposable surface or needle electrodes placed bilaterally on the median nerve at the wrist (upper extremities) and posterior tibial nerve at the ankle (lower extremities). SSEP signals are characterized by latency milliseconds (ms) and amplitude microvolts (µV), with stimulation intensity typically set between 10 and 30 milliampere (mA). SEEPs are monitored to evaluate the function of the dorsal columns of the spinal cord and can be done continuously without interfering with the surgery.

Before the myelotomy, the midline is defined visually. If there are any uncertainties due to deformation caused by the lesion, dorsal column mapping is performed by bipolar stimulation to obtain a tibial SSEP response from the right or left hemisphere. Polarity reversal indicated the midline along the planned myelotomy. In case of significant deterioration in SSEPs during myelotomy, the surgeon is notified, and the myelotomy is either temporarily paused or redirected.

### Motor evoked potentials (MEPs)

Short trains of 5–9 square-wave stimuli (0.5 ms duration per stimulus, with a 3 ms interstimulus interval) are delivered via screw electrodes placed at the C1 and C2 scalp sites, following the international 10–20 EEG system. Single trains of stimulations are performed with intensity ranging from 200 to 1000 V. MEPs are monitored to assess the function of the corticospinal tract.

MEPs were recorded using needle electrodes inserted bilaterally into muscles of both the upper and lower extremities. MEPs were typically recorded from the abductor digiti minimi (upper extremities), tibialis anterior, and abductor hallucis (lower extremities).

MEPs are assessed intermittently to minimize the impact of muscle twitches on microsurgical precision. The loss of MEPs with preserved D-wave signals typically indicates a temporary motor deficit post-operatively. In contrast, the combined loss of MEPs and a reduction of more than 50% in D-wave amplitude is predictive of severe long-term motor deficits [10].

### Direct wave (D-wave)

The D-wave electrode is positioned in the epidural space of the spinal cord, distal (caudal) to the lesion once the spinal canal is exposed. When feasible, an additional electrode is placed proximal (rostral) to the lesion to provide a control recording. A single transcranial electrical stimulus, applied with the same parameters used for MEPs, initiates D-wave recordings. Baseline D-wave measurements are obtained prior to opening the dura. The primary D-wave parameter of interest is amplitude; a reduction of more than 50% from baseline is typically associated with a long-term or permanent motor deficit. In instances where muscle MEPs are lost but D-wave amplitude remains preserved, a transient postoperative motor deficit is generally anticipated. D-waves are monitored to assess the functional integrity of the pyramidal tract and can be performed together with SSEP monitoring continuously without interference with the surgery.

### Interpretation and management

The following IONM alarm thresholds were used: (1) a SSEP amplitude decrease of 50% or a latency increase of 10%; (2) a MEP decrease of 80%; (3) a D-wave amplitude loss of 50%. If any IONM signals passed these alarm thresholds, a standardized response protocol was initiated: (1) halt surgical manipulation; (2) raise the mean arterial pressure (MAP) to 80–90 mm Hg; (3) apply Papaverine to the spinal cord; and (4) pause to allow recovery. If the IONM signals normalized surgery could be resumed. If signal deterioration continued, a team discussion was held to determine whether to proceed or to discontinue the procedure to prevent further neurological injury. If a decreased SSEP amplitude don´t recover but MEPs and D-waves remain stable, the procedure is allowed to proceed. Therefore, decreases in SSEP signals are generally not considered a criterion to abandon surgery, as SSEPs are highly sensitive to surgical manipulation.

## Variables

Outcome measures included motor and sensory function, pain level, bladder and bowel function and, modified McCormic (mMC) score. These measures were assessed both at the short-term 3 months follow-up mark and at long term follow up at least one year after surgery. Preoperative variables assessed included age, sex, intramedullary lesion type (confirmed on histopathology reports postoperatively), degree of functional deficit, presenting symptoms, neurological deficits, affected spinal level, presence of myelomalacia (increased T2 signal on MRI), contrast enhancement of the lesion, and presence of a syrinx. Functional deficits were quantified using the WHO performance score, while neurological deficits were evaluated using the mMC scale.

Intraoperative variables included the extent of visible lesion resection (macroscopic radicality) and intraoperative neurophysiological monitoring (IONM) changes, categorized as unchanged, amplitude decrease, or complete loss of response. Postoperative variables assessed were the radiological degree of surgical radicality achieved, administration of adjuvant radio- or chemotherapy, and mortality.

Outcome measures included assessments of motor and sensory function, pain levels, and bladder and bowel function, as well as modified mMC score. These outcomes were evaluated at a 3-month postoperative follow-up and again at a long-term follow-up, conducted at least one year after surgery.

## Statistical analysis

Categorical data were presented as counts with proportions. For continuous data, the Shapiro–Wilk test was used to assess the normality of distribution. Normally distributed continuous variables are presented as means with standard deviations (± SD), while non-normally distributed variables are presented as medians with interquartile ranges (IQR). The impact of IONM on outcome measures was assessed using odds ratios (95% confidence intervals) derived from logistic regression models. A *p*-value of < 0.05 was considered statistically significant. All statistical analyses were performed using R software (R Foundation for Statistical Computing, Vienna, Austria).

## Results

During the study period, 71 patients with intramedullary spinal lesions underwent surgery with IONM. One patient was excluded due to missing IONM data. The median patient age was 43 years (IQR 30–55), and 61% of the patients were male. The most common lesion types were ependymoma (51%), hemangioblastoma (18%), and cavernoma (8.5%). Lesion location was primarily in the cervical spine (50%), with concomitant myelomalacia present in 67% of cases. The most frequently observed preoperative deficits were sensory impairments (74%), followed by pain (63%) and motor deficits (51%). Additionally, 26% of patients had bladder dysfunction, and 13% had bowel dysfunction. The median duration of symptoms before surgery was 12 months (IQR 6–45).

At presentation, 76% of patients were mMC grade II. Surgical treatment included gross-total resection (GTR, 70%), subtotal resection (STR, 26%), biopsy without further resection (2.9%), and placement of a syringo-subarachnoid shunt (1.4%). On postoperative histopathological analysis, most lesions were WHO grade 1 (41%) or 2 (41%). Adjuvant radiotherapy was administered to 20% of patients, and chemotherapy to 4.3% (high-grade and secondary lesions). Only five patients died during the follow-up period (Table 1). During IONM 31 patients experienced changes in SSEPs, 18 in MEPs, and 5 in distal D-wave responses.

**Table 1:** Description of study population

Variable	Total (*n* = 70)
**Age**	43 [30–55]
**male**	43 (61.4%)
**Lesion type**	
Ependymoma	35 (50%)
Hemangioblastoma	13 (19%)
Cavernoma	6 (8.6%)
Intramedullary lesion (inconclusive PAD)	3 (4.3%)
Astrocytoma	2 (2.9%)
*Other	11 (16%)
**WHO grade**	
1	29 (41%)
2	29 (41%)
3	0 (0%)
4	2 (2.9%)
N/A	10 (14%)
**Preoperative deficits**	
Motor	36 (51%)
Sensory	52 (74%)
Pain	44 (63%)
Bladder	18 (26%)
Bowel	9 (13%)
**Symptom duration (months)**	12.0 [6.00, 45.0]
**Preoperative mMCs**	
1	7 (10.0%)
2	53 (76%)
3	10 (14%)
4	0
**Spinal segment**	
Cervical	38 (54%)
Thoracic	23 (33%)
Lumbar	9 (13%)
**Intramedullary high signal on T2**	47 (67%)
**Surgical radicality**	
GTR	49 (70%)
STR	18 (26%)
Biopsy	2 (2.9%)
Syringo-Subarachnoid Shunt	1 (1.4%)
**Adjuvant radiotherapy**	14 (20%)
**Adjuvant chemotherapy**	3 (4.3%)
**Death**	5 (7.1%)

*Other lesion types composed of 11 unique lesions which included dermoid lesion, diffuse glioma, ependymal cyst, lipoma, lymphoma, PNET, melanocytoma, metastasized adenocarcinoma, schwannoma, subependymoma, and syringohydromyelia

## Postoperative outcomes

**Fig. 1:**
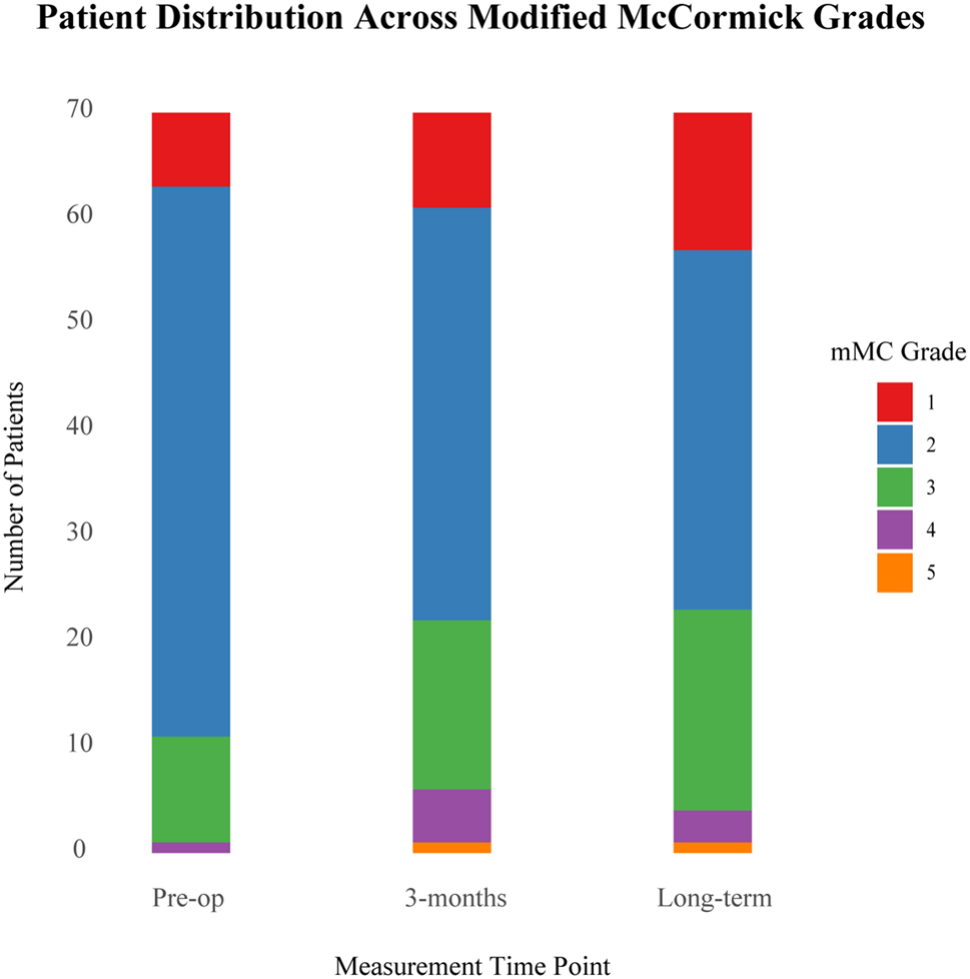
Changes in mMC: Number of patients in each modified McCormic (mMC) grade at pre-operative, 3-months and long-term follow-up

At the three-month follow-up, 92% of patients exhibited some degree of residual deficit. In terms of motor deficits, 21% of patients showed improvement, 43% remained unchanged, and 36% experienced worsening. For sensory deficits, 23% of patients improved, 37% had no change, and 40% deteriorated. Regarding pain, 39% of patients reported improvement, 40% remained stable, and 21% experienced worsening symptoms. In terms of bladder function, 16% of patients showed improvement, 67% remained unchanged, and 17% experienced a decline. For bowel function, 7% of patients improved, 83% remained unchanged, and 10% worsened. For mMC score, 8.9% of patients improved, 69% remained unchanged, and 23% experienced a decline. The long-term follow-up outcomes did not show major changes (Fig. 1, Table 2).

**Table 2:** Postoperative outcomes at 3-month and long-term follow-up

**Outcomes at three months follow-up**						
	Motor deficits	Sensory deficits	Pain	Bowel	Bladder	mMCs
Remaining deficits	45 (64%)	51 (73%)	31 (44%)	10 (14%)	20 (29%)	-
Better	15 (21%)	16 (23%)	27 (39%)	5 (7.1%)	11 (16%)	6 (8.6%)
Unchanged	30 (43%)	26 (37%)	28 (40%)	58 (83%)	47 (67%)	48 (69%)
Worse	25 (36%)	28 (40%)	15 (21%)	7 (10%)	12 (17%)	16 (23%)
**Outcomes at long-term follow-up**						
Remaining deficits	37 (53%)	42 (60%)	35 (50%)	7 (10%)	15 (21%)	-
Better	18 (26%)	20 (29%)	22 (31%)	6 (8.6%)	13 (19%)	10 (14%)
Unchanged	29 (41%)	26 (37%)	27 (39%)	60 (86%)	48 (69%)	41 (59%)
Worse	21(30%)	21 (30%)	21 (30%)	4 (5.7%)	9 (13%)	19 (27%)
Missing	2 (2.9%)	3 (4.3%)	0 (0%)	0 (0%)	0 (0%)	0 (0%)

### Modified McCormic grades

1. Neurologically intact, ambulates normally
2. Mild motor or sensory deficit, patient maintains functional independence
3. Moderate deficit, limitation of function
4. Severe motor or sensory deficit
5. Paraplegic or quadriplegic

## Relationship between IONM and postoperative outcomes

**Fig. 2:**
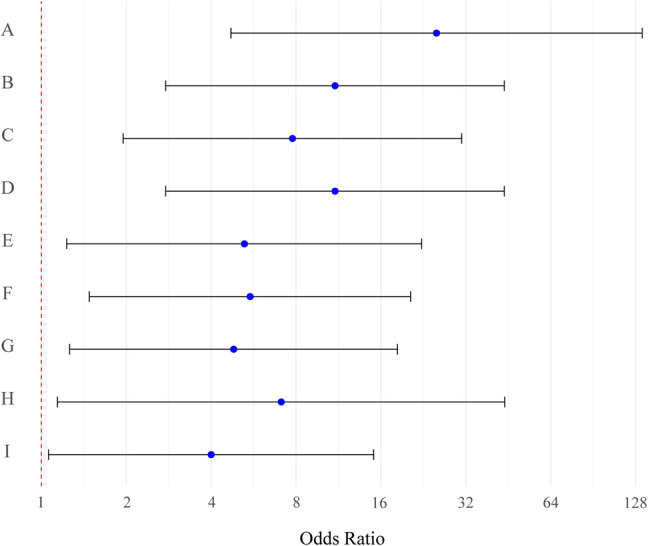
Forest plot showing the odds ratios of all statistically significant predictors. **A**. Sensory deficit at 3 month follow-up/intraoperative loss of SSEP. **B**. Sensory deficit at long-term follow-up/intraoperative loss of SSEP. **C**. Poorer mMC at 3 Month follow-up/intraoperative loss of SSEP. **D**. Poorer mMC at long-term follow-up/intraoperative loss of SSEP. **E**. Pain at 3 month follow-up/intraoperative SSEP amplitude decrease. **F**. Pain at long-term follow-up/intraoperative SSEP amplitude decrease. **G**. Pain at long-term follow-up/intraoperative loss of SSEP. **H**. Motor deficit at long-term/intraoperative MEP amplitude decrease. **I**. Poorer mMC at long-term/intraoperative loss of MEP

Among the 31 patients with intraoperative SSEP changes, 16 had decreased amplitude, and 15 experienced complete loss of response. Decreased SSEP amplitude was associated only with increased pain at the 3-month and long-term follow-ups. In contrast, complete SSEP loss was significantly linked to poorer sensory function in either leg, declines in mMC scores, and increased pain at long-term follow-up (Table 3). At 3 months, patients with intraoperative SSEP loss had 25 times higher odds (95% CI: 4.70–135.07, *p* < 0.001) of sensory deficit in the legs which decreased to 11 (CI: 2.76–43.80, *p* < 0.001) at long-term follow-up. The odds of poorer mMC scores were 7.8 (CI: 1.95–30.96, *p* = 0.004) at 3 months, rising to 11 (CI: 2.76–43.80, *p* < 0.001) at long-term. For pain, a decreased SSEP amplitude raised the odds by 5.3 at 3 months (CI: 1.23–22.32, *p* = 0.025) and by 5.5 at long-term (CI: 1.48–20.39, *p* = 0.011); a complete loss of SSEP intraoperatively increased the odds of pain at long-term by 4.8 (CI: 1.26–18.31, *p* = 0.021) (Table 3, Fig. 2).

**Table 3:** Predictors of postoperative sensory deficits, pain presence, neurologic function, motor deficits, significant amplitude decrease

	3-months follow-up			Long-term follow-up		
Predictor*	OR	95% CI	*p*-value	OR	95% CI	*p*-value
**Postoperative sensory deficit**						
SSEPs amplitude decrease	3.01	0.86–10.59	0.085	3.30	(0.87; 12.53)	0.080
Loss of SSEPs	25.19	4.70–135.07	** < 0.001**	11.00	(2.76; 43.80)	** < 0.001**
**Postoperative pain presence**						
SSEPs amplitude decrease	5.25	(1.23; 22.32)	**0.025**	5.50	(1.48; 20.39)	**0.011**
Loss of SSEPs	4.37	(0.99 to 19.43)	0.052	4.81	(1.26; 18.31)	**0.021**
Postoperative decrease of neurologic function as measured by mMC**						
SSEPs amplitude decrease	1.57	(0.33; 7.52)	0.573	1.27	(0.28; 5.85)	0.76
Loss of SSEPs	7.77	(1.95; 30.96)	**0.004**	11.00	(2.76; 43.80)	** < 0.001**
MEPs amplitude decrease	5.25	(0.89; 30.82)	0.066	4.00	(0.70; 22.88)	0.119
Loss of MEPS	2.63	(0.64; 10.84)	0.182	4.00	(1.06; 15.08)	**0.041**
Postoperative motor deficits						
MEPs amplitude decrease	5.14	(0.84; 31.31)	0.076	7.09	(1.14; 43.96)	**0.035**
Loss of MEPs	2.57	(0.71 to 9.33)	0.151	3.55	(0.95; 13.2)	0.059

*Unchanged signal was considered as the reference for all calculations

**modified McCormick Scale

Among the 18 patients with significant intraoperative MEP changes, 6 experienced a decrease beyond the alarm threshold in MEP amplitude, and 12 had a complete loss of response. A decrease in MEP amplitude was significantly associated with poorer motor function, while a complete loss of MEP response was linked to declines in mMC scores at the long-term follow-up. No statistically significant associations were observed between MEP changes and bladder or bowel function at either short-term or long-term follow-ups. The odds of poor motor function at long-term were 7.1 times higher in patients with a decrease in MEP amplitude (95% CI: 1.14–43.96, *p* = 0.035). Similarly, the odds of a decline in the mMC scores were 4 times higher in patients who experienced a complete loss of MEPs intraoperatively (Table 3, Fig. 2).

Distal D-wave recordings were performed on 39 patients; 34 showed stable responses, 2 showed a significant amplitude decrease and in 3 patients, the D-wave could not be detected at baseline. All five patients had concurrent decreases or loss of MEPs. As only two patients exhibited D-wave changes, we did not perform statistical analysis regarding association of D-wave changes and postoperative outcomes.

## Discussion

The use of IONM is widely recognized as a valuable tool for assessing spinal cord integrity during spine surgeries, supported by Level I evidence [9]. Given that workflows vary between centers, we present outcomes from a workflow used in intramedullary lesion surgery in which a neurophysiologist is present in the operating room to interpret neurophysiological responses in real time. This approach allows the neurosurgical team to perform timely interventions to protect spinal cord integrity during surgery [7]. Continuous monitoring by neurophysiological expertise ensures a high sensitivity and specificity for the detection of potential risks, enabling earlier interventions for safer surgeries [26]. Additionally, objective feedback from IONM enhances patient safety by reducing the likelihood of prioritizing complete resection over neurological preservation [7]. Note however, that surgical removal of intramedullary lesions are high risk procedures where some degree of neurological impairment following surgery may have to be accepted.

In this study, half of the patients exhibited an unchanged degree of neurological deficits, while a third experienced some degree of worsening after surgery. According to the mMC data, only four patients experienced a substantial functional decline. Our results are better than, or in line with, previous reports of about 10% worsening after surgery for intradural lesions [15, 21, 27]. Neurological deterioration following intramedullary spinal surgery can occur due to surgical or vascular insults, some of which may not be detected by standard routine IONM [25].

We found that intraoperative loss of SSEP was the most significant predictor of poor postoperative outcomes, including sensory-motor function and pain. These findings are in line with previous reports in the literature [11, 13, 17, 26]. However, other studies suggest that SSEPs have lower predictive value compared to other modalities for predicting postoperative deficits [11, 22]. Loss of MEP was significant for decline in long-term mMC in this study. The literature supports the value of MEPs as they are associated with better functional outcomes and serve as strong predictors of motor deficits [22, 26]. D-wave monitoring, performed in 39 patients (D-wave monitoring was not available during the entire study period), failed to show significant predictive value in this study, contrasting with existing research where D-waves are often highlighted as the most accurate and reliable modality for predicting long-term neurological outcomes [1, 11]. However, only 2 patients showed a significant amplitude decrease (50, and 60% respectively) but both had unchanged mMC (II) at short- and long-term follow-up.

The outcomes of this study are closely tied to the adherence to the established alarm thresholds in our protocol. These thresholds, are typically based on retrospective data that balance sensitivity and specificity for detecting complications arising from spinal cord manipulation [21]. A 50% decrease in SSEP amplitude or a 10% increase in latency effectively identifies potential disruptions in sensory pathways, with sensitivity values ranging from 85 to 100% and specificity ranging from 43 to 100% [21]. For MEP monitoring, an 80% reduction in amplitude has been shown to predict injuries to motor pathways, with sensitivity ranging from 57 to 100% and specificity from 40 to 100% [21]. While one might argue for a lower threshold, given the high sensitivity of motor neurons to ischemic and mechanical damage, lowering the threshold may increase the risk of false positives [3]. The determination of thresholds for D-wave monitoring remains debated. A 50% reduction in amplitude is commonly associated with damage to descending motor pathways [7]. D-wave monitoring is particularly valuable for predicting long-term motor outcomes, with studies reporting sensitivity between 92 and 100% [1, 11, 29]. The low incidence of functional postoperative deterioration shows that these threshold levels are sufficient for a safe surgery. The available literature regarding what to do when the alarm thresholds are reached suggests two actions. To halt the surgery, temporarily or permanently, and to increase the mean arterial blood pressure [21]. The local application of papaverine has also been suggested in a few studies [19]. The retrospective nature of the material, where all patients were treated according to the same guidelines when indicated, and the lack of a control group precluded any analysis of the efficacy of the interventions per se.

Another important aspect of IONM is the communication between the neurophysiological monitoring team and the rest of the surgical staff in the operating room (OR) [5]. Instances where an assistant neurophysiologist works in several different hospitals with different OR teams, or when an assistant neurophysiologist is present in the OR while a supervising neurophysiologist oversees the data remotely may present challenges to communication [5]. Moreover, effective verbal communication can be compromised by factors such as background noise in the OR or the surgeon needing to focus on particularly demanding stages of the surgery [14, 24, 28]. A setup where the neurophysiologist, rather than the operating surgeon, analyzes the IONM signals lets the surgeon stay focused on the surgical field [19].

IONM is a valuable tool during intramedullary lesion surgery. However, larger prospective studies are needed, comparing gross total resection with an accepted neurological deficit to subtotal resectionSTR, where surgery is stopped because of IONM warning signals. By evaluation of long-term lesion progression and health-related quality of life, evidence could be provided to guide this type of high-risk surgery and clarify the value of IONM warning signals [21].

## Limitations

The primary limitation of this study is its small sample size. Intramedullary lesions are rare, and even with a cohort aggregated over multiple years, it remains challenging to gather enough patients for robust statistical analysis. D-wave monitoring was introduced as routine at the center in 2010 and is a limiting factor to the availability of D-wave data. Another limitation lies in the neurophysiological data, which only categorizes signals as unchanged, decreased in amplitude, or completely lost. More granular data on the type and extent of signal decrease as well as intraoperative dynamic changes over time and after interventions, would be valuable to identify critical thresholds for intraoperative signal changes and correlate them with postoperative neurological outcomes. Similarly, different lesion entities are variably associated with risk for postoperative deterioration depending on histology and exact anatomical location. Statistically adjusting for such information could have been powerful, but our sample size is not sufficient to enable a sensible multivariable analysis.

## Conclusion

The IONM workflow outlined in this study was successfully used in 70 cases of intramedullary spinal cord lesion surgery. Live data from continuous intraoperative neurophysiological monitoring, expertly interpreted in the OR, could potentially be used to make surgical and anesthesiologic adjustments with the goal of minimizing the risk of negative neurological outcomes. Significant associations were found between decreased or lost IONM signals and poorer sensorimotor function and mMC score at short- and long-term follow-up. Implementation of the IONM workflow is suggested in all intramedullary lesion surgery.

## Data Availability

Data is available from the corresponding author upon reasonable request.

## Abbreviations

IONM: Intraoperative neurophysiological monitoring
SSEP: Somatosensory-evoked potentials
MEP: Motor-evoked potential
BCR: Bulbocavernosus reflex
mMC: Modified McMormic
GTR: Gross-total resection
STR: Subtotal resection
D-wave: Direct wave

## Code availability

Code can be provided by the corresponding author upon reasonable request.

## Declarations

### Generative AI and AI-assisted technologies in the writing process

No generative AI was used during the preparation of this work.
